# VP37 Protein Inhibitors for Mpox Treatment: Highlights on Recent Advances, Patent Literature, and Future Directions

**DOI:** 10.3390/biomedicines11041106

**Published:** 2023-04-06

**Authors:** Shuaibu A. Hudu, Ahmed S. Alshrari, Aiman Al Qtaitat, Mohd Imran

**Affiliations:** 1 Department of Basic Medical and Dental Sciences, Faculty of Dentistry, Zarqa University, Zarqa 13110, Jordan; aimanaq@zu.edu.jo; 2 Department of Medical Laboratory Technology, College of Applied Medical Sciences, Northern Border University, Arar 91431, Saudi Arabia; 3 Department of Anatomy and Histology, Faculty of Medicine, Mutah University, Karak 61710, Jordan; 4 Department of Pharmaceutical Chemistry, Faculty of Pharmacy, Northern Border University, Rafha 91911, Saudi Arabia

**Keywords:** VP37 protein, F13L, tecovirimat, NIOCH-14, discovery, development

## Abstract

Monkeypox disease (Mpox) has threatened humankind worldwide since mid-2022. The Mpox virus (MpoxV) is an example of Orthopoxviruses (OPVs), which share similar genomic structures. A few treatments and vaccines are available for Mpox. OPV-specific VP37 protein (VP37P) is a target for developing drugs against Mpox and other OPV-induced infections such as smallpox. This review spotlights the existing and prospective VP37P inhibitors (VP37PIs) for Mpox. The non-patent literature was collected from PubMed, and the patent literature was gathered from free patent databases. Very little work has been carried out on developing VP37PIs. One VP37PI (tecovirimat) has already been approved in Europe to treat Mpox, while another drug, NIOCH-14, is under clinical trial. Developing tecovirimat/NIOCH-14-based combination therapies with clinically used drugs demonstrating activity against Mpox or other OPV infections (mitoxantrone, ofloxacin, enrofloxacin, novobiocin, cidofovir, brincidofovir, idoxuridine, trifluridine, vidarabine, fialuridine, adefovir, imatinib, and rifampicin), immunity boosters (vitamin C, zinc, thymoquinone, quercetin, ginseng, etc.), and vaccines may appear a promising strategy to fight against Mpox and other OPV infections. Drug repurposing is also a good approach for identifying clinically useful VP37PIs. The dearth in the discovery process of VP37PIs makes it an interesting area for further research. The development of the tecovirimat/NIOCH-14-based hybrid molecules with certain chemotherapeutic agents looks fruitful and can be explored to obtain new VP37PI. It would be interesting and challenging to develop an ideal VP37PI concerning its specificity, safety, and efficacy.

## 1. Introduction

**Figure 1:**
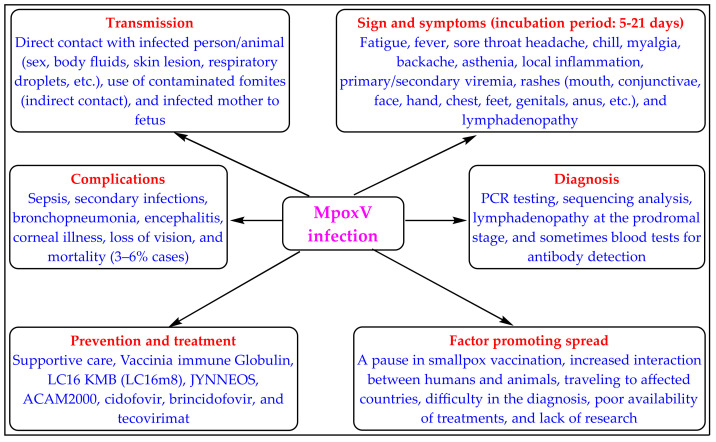
Mode of transmission, symptoms, diagnosis, complications, prevention, and treatments of Mpox.

The zoonotic monkeypox disease (Mpox) is caused by the Mpox virus (MpoxV), which is an enveloped double-stranded DNA virus of the poxiviridae family and the genus Orthopoxvirus (OPV) [1,2,3]. The first case of Mpox was identified in 1958 in monkeys, whereas the first case in humans was reported in 1970 [3]. Mpox is a close relative of smallpox disease caused by another OPV called variola virus [2]. Smallpox was eradicated in the early 1980s, but cases of Mpox have been consistently reported as endemic in African nations. This is because smallpox has humans as the only host, while Mpox has humans and many animals as hosts, making it difficult to eradicate. The mode of transmission, symptoms, diagnosis, and complications of Mpox is depicted in Figure 1 [4,5,6,7,8].

Some outbreaks of Mpox were reported in non-African countries such as the United States of America (2003 and 2021), the United Kingdom (2018), Northern Ireland (2021), and Singapore (2019) [1,3]. However, the Mpox outbreak of 2022 has caused a serious health concern worldwide. Until 25 January 2023, the WHO reported 85,106 confirmed cases and 83 deaths in 110 countries [9]. There are two clades of MpoxV, wherein clade IIb is considered the major cause of the Mpox outbreak of 2022 (Table 1) [10].

Even though the globe is currently grappling with the pandemic instigated by the coronavirus disease 2019 (COVID-19), the Mpox outbreak has caused public health experts to express alarm about the possibility that it could pose a new global threat [11].

Tecovirimat is an approved treatment for Mpox [12]. The Centers for Disease Control and Prevention (CDC) has also recommended some antivirals and vaccines to battle Mpox on a need basis [13] (Table 2).

## 2. Drug Targets and VP37 Protein (VP37P)

Vaccinia virus is the prototype of OPVs and has an established replication cycle. Accordingly, numerous Vaccinia virus-based molecular drug targets for developing anti-OPV drugs have been reported in the literature (Table 3) [3,10,14,19,20].

Because of the similarity in the viral genome of OPVs, especially in the VP37 protein (VP37P), the treatments already available for smallpox might also be successful for Mpox. Tecovirimat has the most compelling evidence supporting its efficacy and safety in treating Mpox [3,21]. As a result, developing novel antivirals focused on specific targets such as the VP37P can increase the accessibility and range of effective anti-OPV drugs. This article spotlights the existing and prospective VP37PI-based treatments for Mpox and other OPV infections.

The updated literature (patent and non-patent) for this manuscript was collected on 17 January 2023, from PubMed and different free patent databases (Espacenet, Patentscope, and USPTO) utilizing various keywords (VP37, p37, F13L, Mpox, smallpox, Orthopoxvirus, tecovirimat, TPOXX, ST-246, SIGA-246, NIOCH-14, and IMCBH) and their combinations [3,14]. The identical results were removed, and the literature relevant to the subject matter was selected for writing this article. Our patent and non-patent searches provided only a few specific VP37P inhibitors (VP37PIs). Many patents/applications related to modified viruses/F13L genes were identified for preparing vaccines for OPV infections. Accordingly, those patents/applications are not discussed in this review.

**Figure 2:**
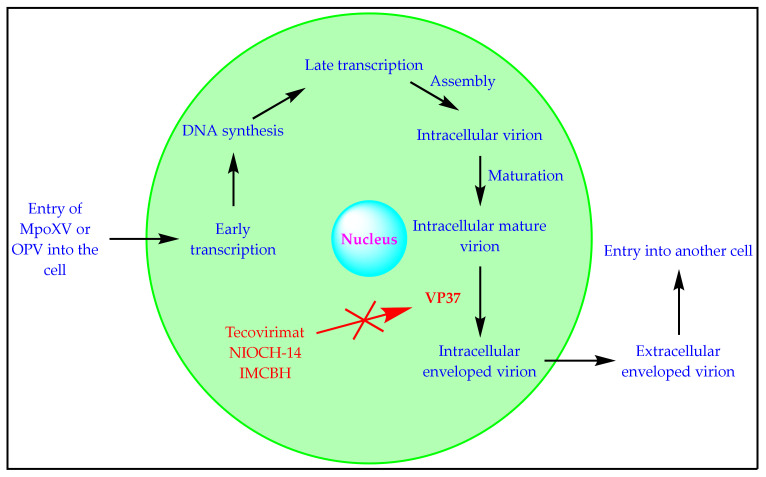
The replication cycle of an OPV/MpoxV.

MpoxV and other OPVs are DNA viruses that replicate in the cytoplasm instead of the nucleus of the infected cells. For a virus to strive and cause infection, it must effectively recognize, penetrate, uncoat, and synthesize all the required proteins needed to form a matured virus. The replication cycle of an OPV is provided in Figure 2 [3,14,19,20,22].

The proteins of OPVs are synthesized from the viral genome’s open reading frames (ORFs). Table 3 lists the genes responsible for the synthesis of OPV proteins. Mpox and other OPVs have an established viral genome [19,20,22]. Most of these ORFs are conserved for all OPVs, for instance, F13L, which encodes for the VP37P (37 kDa) of OPVs [23]. Interestingly, the E353K alteration in the VP37P was identified in the leakage responsible for the 2022 Mpox outbreak [12]. This protein is required for the viral interaction with human target cell membrane protein TIP47 and Rab9 of infected cells, catalyzing the intra-cellular viral particle maturation to competent variola viral particles and the subsequent assembly of the extracellular virus [23,24,25].

## 3. Marketed and Patented Drugs 

### 3.1. Tecovirimat (TPOXX, ST-246, and SIGA-246)

**Figure 3:**
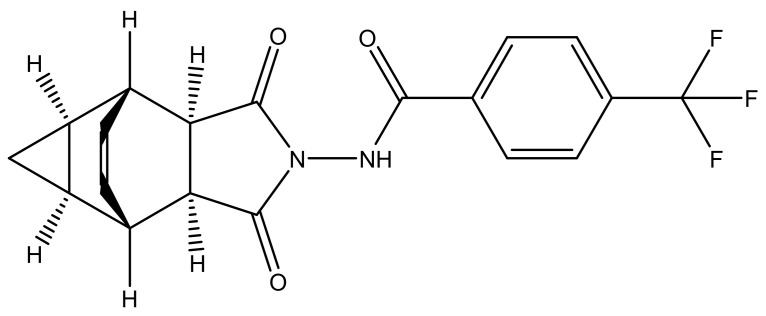
Chemical structure of tecovirimat.

Tecovirimat, a tetracyclic acylhydrazide derivative, was first identified as an anti-OPV agent in 2002 [3] (Figure 3), whereas WO2004112718A2 was the first published patent application claiming tecovirimat as an anti-OPV infection agent [26].

Tecovirimat inhibits the VP37P-based formation of the envelope surrounding OPVs, including MpoxV. The formation of this envelope is crucial for the virus to escape the cell and infect other cells. Accordingly, tecovirimat prevents the OPV/MpoxV from spreading to other cells in the body (Figure 2) [3,19,27]. It is pertinent to know that tecovirimat does not stop the production of matured viruses within the infected cells and does not prevent DNA/protein synthesis. Still, it stops the propagation of the virus within a host that already has it, as well as the virus’s ability to infect other hosts [3,19,21]. Tecovirimat is approved by the European Medicine Agency (EMA), the United States Food and Drug Administration (USFDA), and Health Canada for the treatment of different OPV infections [28,29,30]. It is important to mention that human OPV-based clinical trials (CTs) are neither possible nor ethical. The effectiveness of tecovirimat has been confirmed in diseased rabbits and non-human primates following the Animal Rule set forth by the USFDA [31]. Tecovirimat’s important in vitro and in vivo activity data are available in the literature [3]. The important information on the approved tecovirimat (TPOXX) is provided in Table 4.

It was demonstrated that the dosage of tecovirimat that was 50% effective was less than 0.04 µM [34]. If treatment with tecovirimat was started for up to eight days after a potentially fatal MPXV exposure, it increased the number of patients who survived the illness. Once given five days after the virus exposure, the medication was proven to help protect against clinical illness and offer some degree of protection [35]. The patent literature on tecovirimat is provided in our previous article [3]. Therefore, herein, we summarize only a few important patents/patent applications of tecovirimat.

US11433051B2 claims a dry suspension of crystalline polymorphic form I of tecovirimat and simethicone, which may further contain other pharmaceutically acceptable excipients such as a suspending agent, lubricant, antifoaming agent, sweetener, and a flavoring agent [36]. It also claims a method of treating OPV infections and eczema vaccinatum utilizing a dry suspension of crystalline polymorphic form I of tecovirimat [36]. 

US8642577B2 claims a synergistic combination of cidofovir/brincidofovir and other antiviral drugs such as tecovirimat for treating OPV infections, including Mpox, smallpox, cowpox, mousepox, rabbitpox, and camelpox [37].

EP2202297B1 claims the use of an antiviral agent (tecovirimat, cidofovir, brincidofovir, and imatinib) or a combination thereof in the preparation of a medicament for treating an adverse side effect (pock formation, weight loss, fever, abdominal pain, aches or pains in muscles, cough, diarrhea, and feeling of discomfort or illness) associated with the oncolytic pox virus (vaccinia virus) therapy of cancer (metastatic cancer and a solid tumor) [38].

CN115141136A claims a co-crystal comprising tecovirimat and a co-crystal ligand (p-hydroxybenzoic acid, benzoic acid, isonicotine, nicotinamide, acetamide, benzamide, piperazine, or monoethanolamine), wherein the molar ratio of tecovirimat co-crystal ligand is 1:0.5–5. It also claims a pharmaceutical composition of the claimed co-crystal with a pharmaceutically acceptable carrier/excipient or vaccine for treating OPV infections [39].

### 3.2. NIOCH-14

**Figure 4:**
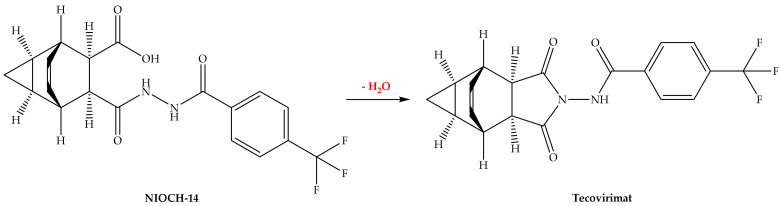
Metabolism of NIOCH-14.

NIOCH-14, a water-insoluble tricyclo-dicarboxylic acid derivative, is a prodrug of tecovirimat. NIOCH-14 is cyclized in the human body to provide tecovirimat as the active metabolite (Figure 4) [40]. 

**Figure 5:**
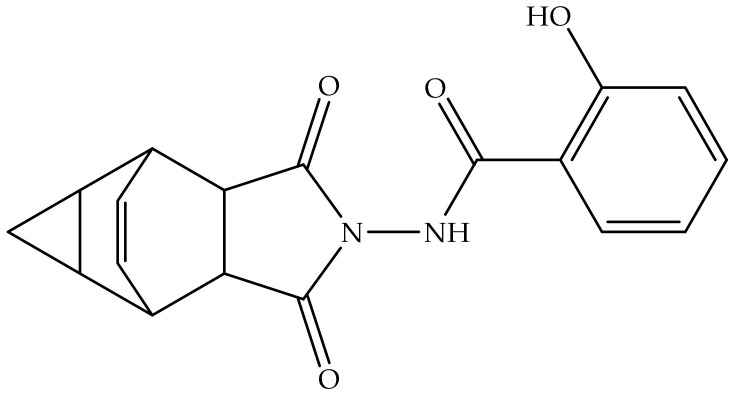
Chemical structure of NIOCH-32.

NIOCH-14 was first disclosed in 2009 as an anti-OPV drug and received patent certification in 2011 in Russia as RU2412160C1 [41]. RU2412160C1 mentions anti-OPV data of NIOCH-14. However, the authors did not properly understand the English translation of this Russian patent. Another published article provides important comparative in vitro anti-OPV activity data for NIOCH-14, tecovirimat, and NIOCH-32 (Figure 5), which are summarized in Table 5 [42].

A comparative study of NIOCH-14 with tecovirimat in mice lungs infected with MpoxV (V79-1-005) showed positive and similar efficacies after seven days (oral dose 30 µg/g and 60 µg/g of mouse weight) [43]. The activity of NIOCH-14 was slightly better than tecovirimat. Similar observations were reported in another study [42]. Additional comparative study of NIOCH-14 with tecovirimat in mice lungs infected with variola virus (strain India-3a) revealed that both drugs lowered the variola virus concentration to a similar extent in lungs after four days of the treatment (oral 50 µg/g) [44]. NIOCH-14 also displayed a better bioavailability of 22.8% than tecovirimat (12.1%) in a mice model (single dose of 50 µg/g) [45]. Similarly, in a recent study, the efficiency of NIOCH-14 towards OPV, except for the variola virus, was reported to be safe and bioavailable at a dose of 5 g/kg in experimental animals [46].

The LCMS-based therapeutic drug monitoring clinical study data of protocol number NIOCH-01/20 have been published [40]. NIOCH-14 metabolizes quickly in blood to its primary metabolite (tecovirimat). Therefore, the pharmacokinetic parameters of NIOCH-14 (single oral dose = 600 mg) were assessed based on the concentration of tecovirimat in the blood (Table 6). It has been stated that a 600 mg dose of NIOCH-14 is equivalent to 250 mg tecovirimat [40].

According to the World Health Organization’s report, NIOCH-14 has a similar mechanism of action to tecovirimat (VP37PI); NIOCH-14 has passed the clinical phase I trial in Russia (oral capsule 200 mg; 90 participants of 18 to 50 years; protocol number NIOCH-01/20); the clinical phase II/III may finish by 2023–2024, and NIOCH-14 may receive marketing authorization by 2023–2024 [47].

The patent literature search provided many NIOCH-14-based granted patents in Russia. These Russian patents are summarized below. 

RU2543338C1 claims the therapeutic and prophylactic use of NIOCH-14 (once daily over a dose range of 4 to 60 mg/kg of mammal body weight for four days) to treat smallpox [48]. The description of RU2543338C1 confirms the antiviral activity of NIOCH-14 in variola virus-infected mice. It also confirms the antiviral activity of NIOCH-14 against surrogate OPVs (vaccinia virus, cowpox, and ectromelia) and a synergistic effect with tecovirimat [48].

RU2716709C1 claims a capsule dosage from the treatment and prevention of diseases caused by OPVs, wherein the capsule contains NIOCH-14 (180–220 mg), lactose monohydrate, silicon dioxide, magnesium stearate, and microcrystalline cellulose (MCC) [49]. It is imperative to note that the clinical trial NIOCH-14 has been completed in capsule dosage form [47].

RU2542490C1 claims the use of NIOCH-14 for stopping unwanted post-vaccination reactions and complications of smallpox vaccines, wherein oral NIOCH-14 (3.3–50 mg/kg) is administered as a single dose for three days starting from the day of vaccination [50].

### 3.3. N1-Isonicotinoyl-N2-3-methyl-4-chlorobenzoylhydrazine (IMCBH)

**Figure 6:**
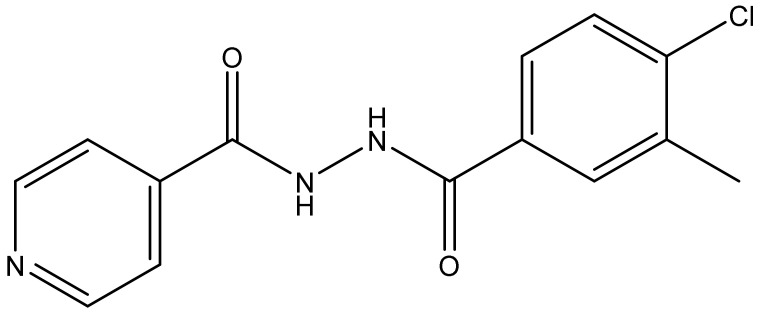
Chemical structure of IMCBH.

IMCBH, an isonicotinohydrazide derivative, inhibits the F13L gene/VP37P of OPVs and prevents the secondary envelopment and extracellular enveloped virion formation of OPVs similar to tecovirimat [19,21] (Figure 6).

IMCBH’s selective vaccinia virus multiplication blocking property and virus release inhibitory effects (3 µg/mL) were reported in 1969. However, IMCBH did not inhibit the intracellular formation of the vaccinia virus [21,51]. These findings were further confirmed in 1979 and 1981 [52,53]. Another study published in 1991 indicated that IMCBH produces its antiviral effect by inhibiting the 37K protein encoded by the F13L gene, and a mutation of the 37K envelope protein of the vaccinia virus imparts drug resistance to IMCBH [54]. Interestingly, IMCBH was found to be active in vitro studies, but it did not demonstrate protection in mice and rabbits [21,51].

Our patent search provided a few IMCBH-based inventions. US8642577B2 claims a synergistic combination of cidofovir/brincidofovir and other antiviral drugs such as IMCBH for treating OPV infections, including Mpox, smallpox, cowpox, mousepox, rabbitpox, and camelpox [37]. This patent does not provide the experimental details of the combination of cidofovir/brincidofovir and IMCBH [37]. WO2013165898A2 claims 1,8-naphthyridine derivatives as inhibitors of the resolvase enzyme of OPV, which can be combined with IMCBH and other anti-OPV agents to treat OPV infectious diseases [55].

## 4. Discussion

OPV-based infections such as smallpox have been recognized as a possible source of a bioterrorism attack. Accordingly, the USFDA planned strategies to develop drugs for OPV-induced infections [3]. Mpox is an OPV-induced infection that has threatened humankind worldwide since mid-2022 [10]. OPVs share almost similar genomic structures. Therefore, the drugs and vaccines developed for smallpox can also be effective for Mpox [19,21]. The current possible treatments for Mpox are mentioned in Table 2.

Mpox infection was linked with abnormal Mpox-related genes (MpoxRGs) expression, which significantly correlated with tumor immunology and the drug response pathway when activated [56]. This MpoxRGs was found to be expressed differently in varieties of tumor cells and was also used as prognostic markers in tumors [57]. MpoxRGs tend to be elevated in tumors and express themselves differently depending on the category and grade of the tumor. Mpox might be capable of influencing carcinogenesis, according to a survival analysis that revealed that a high MpoxRG score was often strongly related to a bad tumor prognosis [57]. Similarly, a genetic study showed that MpoxRG copy quantity and single nucleotide variants were related to tumor longevity [56]. MpoxRG alterations were significantly correlated with survival, indicating that tumor prognostic may be impacted by polymorphisms [57].

Many drug targets or developing anti-OPV infections have been identified (Table 3) [10]. Among these drug targets, OPV-specific VP37P (encoded by F13L gene/protein) is an established drug target for developing drugs for OPV-based infections, including Mpox. One VP37PI (tecovirimat) has already been approved in Europe to treat Mpox, while another drug, NIOCH-14, is under clinical trial. 

Tecovirimat and NIOCH-14 have similar chemical structures and mechanisms of action (Figure 3). A recent publication has spotlighted the importance of adamantane derivatives for discovering the inhibitors of VP37P [58]. The authors opine that developing the adamantane-based hybrid molecules of tecovirimat/NIOCH-14 may also possess activity against VP37P. Some adamantane-based chemotherapeutic agents (amantadine, rimantadine, tromantadine, adaphostine, adarotene, opaganib, and SQ109) are reported [59]. The development of tecovirimat/NIOCH-14-based hybrid molecules with these chemotherapeutic agents looks fruitful and can be explored to obtain a new VP37PI. 

Natural products are a good source of identifying lead compounds and developing new drugs [60]. Many natural remedies have been suggested to display activity against Mpox, including *Acacia nilotica*, *Adansonia digitata*, *Aframomum melegueta*, *Allium sativum*, *Anogeissus leiocarpus*, *Azadirachta indica*, *Balanites aegyptiaca*, *Boscia senegalensis*, *Calotropis procera*, *Carica papaya*, *Cassia singueana*, *Citrullus lanatus*, *Cucurbita maxima*, *Diospyros mespiliformis*, *Ficus platyphylla*, *Ficus polita*, *Guiera senegalensis*, *Lawsonia inermis*, *Mangifera indica*, *Maytenus senegalensis*, *Moringa oleifera*, *Nigella sativa*, *Olea europea*, *Piper guineense*, *Sterculia setigera*, and *Momordica charantia* [61]. However, only a few experimental studies have been carried out on anti-Mpox activity and the mechanism of action of the phytoconstituents of these plants. A molecular docking-based study on the phytoconstituents of these plants to identify VP37PIs may provide lead compounds for Mpox. 

Virus mutation causes drug resistance and necessitates discovering new therapeutic agents and treatments. Mutations in the F13L, D13L gene, and VP37P have been recognized [3,19,21,62] with the emergence of viral resistance to rifampin, a drug that interacts with D13L previously employed for the treatment of OPV infection [62]. The mutation to the D13L of the glycoprotein-27 interferes with the bond between the D13L and previously effective drugs such as simeprevir and rifampin, leading to the development of resistance due to the loss of the persistent peptide bond. This resistance affects both clades of the MpoxV as the drug targets are the same for both clades. Therefore, there is a need for an effective synthetic or repurposed drug, as the current Mpox treatments may become less effective against such mutants. When dealing with patients not responding to tecovirimat, the danger of resistance must be considered [20]. Therefore, the availability and range of potent anti-Orthopoxvirus medicines and a synergistic drug combination reduce the development of drug resistance and can also shorten the therapy duration [10]. 

The replication cycle of an OPV involves various steps (Figure 2). The inhibitors of the different stages of the replication cycle of OPVs have been reported [10,19,21]. Tecovirimat and NIOCH-14 are VP37PIs. A combination of tecovirimat/NIOCH-14 and the drugs affecting the various stages of the replication cycle of OPVs, including DNA ligase inhibitor (mitoxantrone), topoisomerase inhibitors (ofloxacin, enrofloxacin, and novobiocin), DNA polymerase inhibitors (cidofovir and brincidofovir), Tyr/ser protein phosphatase inhibitor (ethacrynic acid), late-transcription elongation factor inhibitor (methisazone), early viral transcription inhibitors (adenosine N1-oxide and nigericin), egress inhibitors (terameprocol and imatinib), and rifampicin may produce better therapeutic outcomes for Mpox treatment. Idoxuridine, trifluridine, vidarabine, fialuridine, and adefovir have also displayed anti-OPV activity. The combination of these drugs can also be assessed with tecovirimat and NIOCH-14. The assessment of the synergistic effects of some common anti-Mpox natural remedies (garlic, *Nigella sativa* oil, moringa oil, and olive oil) and immunity boosters (vitamin C, zinc, thymoquinone, quercetin, ginseng, etc.) is also recommended [61]. However, attention must also be paid to drug–drug interactions (chemical and pharmacological) while developing new combinations of existing VP37PIs with other drugs. 

Drug repurposing is an effective strategy to develop essential medicine quicker than the conventional drug discovery process [63]. The F13L gene encodes VP37P. A study has demonstrated similar interaction of fludarabine (an established DNA-dependent RNA polymerase inhibitor as an anticancer drug) and tecovirimat with F13L protein, suggesting the possible use of fludarabine for Mpox [64]. Fludarabine is a purine analog. Many other purine analogs in clinical practice have a structure similar to fludarabine, such as clofarabine, cladribine, and nelarabine [65,66]. These drugs may be assessed for their VP37/F13L protein inhibitory activity.

The cessation of the smallpox vaccination is one of the reasons for the spread of Mpox. The smallpox vaccination has shown protection against Mpox. However, the smallpox vaccine has certain side effects. A patent claiming NIOCH-14 (a VP37PI) to prevent the side effects of the smallpox vaccine has been published in Russia [50]. Similarly, comments have been made on tecovirimat [67]. This indicates that VP37 may also be useful to cease or reduce the side effects of smallpox vaccines. However, drug–vaccine interactions need further investigation to establish the safe and effective combined use of tecovirimat/NIOCH-14 among different patient populations (pediatric, geriatric, pregnant women, immune-compromised patients, etc.).

There is a shortage in the discovery and development of VP37PIs. This shortcoming makes developing VP37PIs an interesting area for further research to develop anti-OPV agents, including anti-Mpox therapeutics. However, it would be interesting and challenging to develop an ideal VP37PI concerning its specificity, safety, efficacy, potency, tolerability, chemical stability, oral bioavailability, and patient-compliant dosage forms/dosing regimen that does not require medical supervision and has no interaction with the vaccine.

## 5. Conclusions

The OPV-specific VP37 protein (VP37P) is an important target for developing drugs against OPV-based infections, including Mpox. However, very little work has been carried out on developing VP37 protein inhibitors (VP37PIs). This aspect increases the scope of further research in developing VP37PIs. Developing tecovirimat/NIOCH-14-based hybrid molecules with the existing clinically used DNA polymerase inhibitors may provide new chemical templates with a dual mechanism of action. Drug repurposing seems to be a good strategy for identifying clinically useful VP37PIs. Developing new combination therapies of the existing VP37PIs (tecovirimat and NIOCH-14) also appears promising to fight against Mpox and other OPV infections.

## Figures and Tables

**Table 1:** Clades of MpoxV.

Clade (Synonym)	Subclade	Affected Countries	Mortality Rate
I (Congo Basin or Central African clade)	-	Central Africa, Cameroon, Congo, Gabon, South Sudan	10.6%
II (West African clade)	IIa	Liberia, Nigeria, Sierra Leone, Cameroon, Cote d’Ivoire	3.6%
	IIb	Main clade for Mpox 2022 outbreaks	

**Table 2:** Treatment and prevention of Mpox.

Drug Name	Drug Category	Mechanism of Action	Reference
Tecovirimat	Antiviral	VP37 protein inhibitor (VP37PI)	[3]
Cidofovir	Antiviral	DNA polymerase inhibitors	[14]
Brincidofovir (Prodrug of cidofovir)	Antiviral	DNA polymerase inhibitors	[14]
Vaccinia immune globulin	Blood product	Kills mature virus or intracellular mature virus	[13,15]
LC16 KMB (LC16m8)	A third-generation, live, replicating attenuated vaccine containing live vaccinia virus (LC16m8 strain)	Neutralizes the Orthopoxvirus infection-induced antibodies	[16]
JYNNEOS	An attenuated, live, non-replicating, third-generation vaccine	Stimulates cellular and humoral immunity against Orthopoxvirus infection-induced antibodies	[13,17,18]
ACAM2000	ACAM2000 is the second generation live attenuated vaccine containing live vaccinia virus	The vaccine stimulates a person’s immune system to produce antibodies against OPVs	[13,17,18]

**Table 3:** Molecular targets for developing anti-OPV drugs [3,10,14,19,20].

S. No	Stage of Replication	Examples of Inhibitors	Drug Target (Gene)
1	DNA processing and packaging	Mitoxantrone (DNA ligase); ofloxacin, enrofloxacin, and novobiocin (topoisomerase type IB)	DNA helicase (A18R), Holliday junction resolvase (A22R and K4L), DNA ligase (A50R), topoisomerase type IB (H6R), telomere binding proteins (I1L, I6L, and K4L)
2	DNA replication	Cidofovir, brincidofovir, aphidicolin, cytosine arabinoside, and phosphonoacetic acid (DNA polymerase); siRNA and small peptide aptamers (NTPase)	Polymerase processivity factor (A20R and D4R), uracil DNA glycosylase (A20R and D4R), NTPase (D5R), DNA polymerase (E9L), Substrate of B1R kinase (H5R), ssDNA-binding phosphoprotein (I3L)
3	Enzymatic targets	Hydroxyurea (ribonucleotide reductase subunits); aurintricarboxylic acid and ethacrynic acid (tyr/ser protein phosphatase); TTP-6171 (essential viral proteinases); siRNA (Ser/Thr kinase); 5-iodo-4′-thio-2-deoxyuridine and N-methanocarbathymidine (thymidine kinase)	Thymidylate kinase (A48R), ser/thr kinase (B1R), ribonucleotide reductase subunits (F4L and I4L), essential ser/thr kinase (F10L), tyr/ser protein phosphatase (H1L), essential viral proteinases (I7L and G1L), thymidine kinase (J2R)
4	Entry and uncoating	-	Structural and membrane proteins (A16L, A17L, A21L, A26L, A27L, A28L, B5R, D8L, F9L, G3L, G9R, H2R, H3L, J5L, L1R, and L5R)
5	Morphogenesis	Rifampicin (rifampicin resistance protein)	Assembly complex (A10L, A15L, A30L, D2L, D3R, F10L, F13L, G7L, and J1R), nonstructural proteins (A11R and A32L), rifampicin resistance protein (D13L), intracellular mature virus (H3L), intracellular mature virus membrane protein (L1R)
6	Transcription and mRNA processing	Isatin-β-thiosemicarbazones and methisazone (Late-transcription elongation factor; Poly(A) polymerase Subunit; RNA polymerase subunits)	RNA polymerase subunits (A24R, A29L, A5R, D7R, E4L, G5.5R, H4L, J4R, and J6R), NPH-I (D11L), mRNA capping enzyme (D12L, D1R), Poly(A) polymerase VP55 and V39 (E1L, J3L), late-transcription elongation factor (G2R), RNA polymerase-associated protein (RAP94) (H4L)
7	Formation of extracellular enveloped virus	Tecovirimat, NIOCH-14, and IMCBH (VP37 protein); vaccinia immune globulin (Extracellular enveloped virion membrane glycoprotein)	Intracellular mature virus surface protein (A27L), intracellular enveloped virions transmembrane protein (A36R), extracellular enveloped virion membrane glycoprotein (B5R), actin tail formation (A33R and (A34R), extracellular enveloped virus formation protein called VP37 (F13L)

**Table 4:** Important information on marketed tecovirimat [3,30,32,33].

Parameter	Summary
Innovator	Siga Technologies Inc. (United States)
Chemistry	Marketed form: water-insoluble and non-hygroscopic crystalline tecovirimat monohydrate (C_19_H_15_F_3_N_2_O_3_·H_2_O); molecular weight: 394.35; CAS registry number: 1162664-19-8; BCS class: II
Approval date (Dosage forms)	USFDA: 13 July 2018 (capsule, 200 mg); 18 May 2022 (intravenous solution, 10 mg/mL); EMA: 6 January 2022 (capsule, 200 mg); Health Canada: 29 November 2021 (capsule, 200 mg)
Indications	USFDA: smallpox; EMA: Mpox, smallpox, cowpox, and vaccinia virus infections; Health Canada: smallpox
Pharmacokinetic parameters	T_max_ (600 mg p.o.): 4–6 h; volume of distribution (600 mg p.o.): 1030 L; major metabolites: glucuronic acid conjugate; main route of elimination: urine (73%); half-life (600 mg p.o.): 19 h; clearance (600 mg p.o.): 31 L/h; LD_50_ (mice/non-human primates): 2000 mg/kg

**Table 5:** In vitro anti-OPV data of NIOCH-14 [42].

Compound	50% Toxicity Concentration (µg/mL)	IC_50_ in µg/mL			Therapeutic Indices
		V79-1-005 Strain of Mpox	Butler, Congo-9, and India-3a Strains of Variola Virus	Ectromelia Virus Strain K-1	
NIOCH-14	>100	0.013	0.001–0.004	0.011	>100,000
Tecovirimat	>100	0.003	0.001–0.004	0.003	>100,000
NIOCH-32	>100	0.153	0.032–0.078	0.54	>3100

**Table 6:** Pharmacokinetic parameters of NIOCH-14 and tecovirimat [46].

Parameters	NIOCH-14		Tecovirimat	
	IV (2 µg/g)	Oral (50 µg/g)	IV (2 µg/g)	Oral (50 µg/g)
Half-life (T_1/2_, hours)	2.3	5.7	2.0	3.4
Time of maximum concentration (T_max_, hours)	0.25	6	0.25	3
Maximum measured concentration (C_max_, ng/mL)	9515 ± 3903	15,439 ± 3373	13,200 ± 4287	15,495 ± 3227
Area under the curve (AUC_0-t_, ng/(mL/h))	24,918	141,883	34,254	103,661
Area under the curve (AUC_0-inf_, ng/(mL/h))	25,038	142,220	34,318	105,405
Absolute bioavailability (%)	-	22.8	-	12.1

## Data Availability

The data provided in this manuscript is available in the cited references.
